# Pandemic fatigue amidst COVID-19: Are we slacking off?

**DOI:** 10.1016/j.amsu.2022.104356

**Published:** 2022-08-18

**Authors:** Hamna Raheel, Aiman Rahim, Unaiza Naeem

**Affiliations:** Department of Internal Medicine, Dow University of Health Sciences, Karachi, Pakistan

On 1st March 2019, Pakistan saw its first Covid-19 case. Three years, three variants, many lockdowns, and 30413 deaths later, we are still captured in fear of deteriorating health and a failing healthcare system [1].

The World Health Organization (WHO) declared COVID-19 a pandemic in 2020 after its spread to 190 nations [2]. Research has shown that a new human infectious disease emerges every eight months approximately. The 1918 flu fatigue and the Asian flu of 1957 have been significant adversities in the past. More than 35 emerging infectious diseases have been seen since 1980. The vulnerability of the human population to diseases and the increasing resistance to microbes has many lessons to learn from [3,4].

Pandemic fatigue is a natural phenomenon that occurs over time because of a prolonged health crisis. It is demotivation to follow recommended protective behaviors, emerging gradually and affected by several emotions, experiences, and perceptions [5]. COVID-19 is also an occurrence now marked by pandemic fatigue. A befitting way to explain such an event is through cognitive control [6].

Simply put, it is a cost-benefit analysis; due to ever-changing behavioral guidelines, our mind weighs the value we get from performing a specific task and the cost we pay for it. Another factor that plays a role in the mental effort is that the pandemic has forced us to multitask and balance the fine line between work and life. The sheer longevity of this recent pandemic and the economic burden incurred consequently have led people to undermine the risk of the virus. COVID-19 has had an unprecedented impact on the daily lives of people. Individuals face a continuous cognitive challenge regarding day-to-day activities such as commuting, shopping, and leisure activities that require a new set of social protocols to be followed. These safety guidelines often discount the value we get and lead to undesirable outcomes, such as reduced visits to family members and financial hardships due to businesses closing.

Complacency regarding COVID-19 protective guidelines and the severity of the virus has led to a surge in cases. Most citizens often avoid getting tested; this makes it increasingly challenging to identify hotspot areas that serve as breeding grounds for COVID cases. In addition, low testing rates have led to inflated positivity rates, which do not accurately represent COVID-19 infection rates. Various other factors can be attributed to non-compliance with health protocols. A qualitative study from Iran found that mental barriers, including insufficient knowledge and lack of understanding of the severe threat to their life, were associated with nonchalance towards regulations [7]. Other barriers listed were emotional ones, such as depression from quarantine and a decline in familial relations that has resulted in a lack of motivation. Economic losses have also led people to disregard safety measures and work despite being sick. Lack of equal and accessible resources and treatment and inadequate sound awareness by the media have also made people disillusioned [7].

Another detrimental effect of pandemic fatigue is a decreased allocation of resources for the COVID-19 vaccine rollout and pandemic relief. The government abolished the NCOC (National Command of Operation and Control) when infection rates started to decline. Some of the duties of this commission included overseeing COVID-19 relief efforts and designing campaigns for media outlets regarding SOPs [8].

To dispel pandemic fatigue and its consequent effects, some of our practices part of the “new normal” need to be ameliorated. While lockdown policies were effective in the first half of the pandemic due to limited knowledge and treatment options, they appear ineffective in the long run. Identifying low-risk and harm-reducing strategies to modify daily activities has proven to be a better approach than a complete halt on daily activities. A sustainable way is to focus on lowering risk and engaging people to find creative ways to deal with the problems. In Israel, a business opened a floating cinema where people could enjoy a film on a boat all while following COVID-19 guidelines. Mass gatherings have changed to a hybrid model, with fewer gatherings outside and more gatherings online to accommodate the same number of people.

WHO has proposed four principles to reinvigorate the public; (i) understanding people, (ii) engaging with the public regarding plausible solutions, (iii) allowing people to live their lives while ensuring minimal risk, and (iv) acknowledging the hardships people face [9]. Researchers should perform qualitative and quantitative research on the public's perceptions regarding restrictions. One example is Romania, where a behavioral survey was used to devise policy regarding reopening schools. Ukraine used similar surveys to identify critical barriers in compliance with Covid-19 protocols and used the results to target the concerns of those non-compliant towards the restrictions [5].

Engaging the public and involving them in COVID-19 prevention policies is essential to remove mistrust and overcome the disconnect we see between the government and the public. One way of garnering public trust is to bring community leaders such as religious scholars or prominent public figures in the loop. During the Ebola epidemic, Christian and Muslim scholars approved the modified ways of burying those infected by the disease to prevent the spread of Ebola [9]. In Pakistan, an Islamic Advisory Group on Polio Eradication trained renowned religious scholars to debunk religious misconceptions among families hesitant about the polio vaccine [10].

COVID-19 continues to present new challenges and as we grapple with the repercussions of the virus, we must also be cognizant of the effects observed as preventive measures are placed to be followed by the masses, one such manifestation being the pandemic fatigue. Overcoming the gap between the policy makers and public is important for ensuring that sustainable options are considered when devising contingency plans during the pandemic, that are heedful of the populace's needs and opinions. It is imperative to develop regulations that not only limit the spread of the virus but also help in restoring a feeling of normalcy in people's daily lives, meanwhile allowing them a facile transition into adapting to the changes in their routines brought about by the pandemic.

## Ethical approval

This paper did not involve patients, therefore no ethical approval was required.

## Source of funding

None.

## Author contribution

Hamna Raheel = conception of the study, drafting of the work, final approval and agreeing to the accuracy of the work, Aiman Rahim = conception of the study, drafting of the work, final approval and agreeing to the accuracy of the work, Unaiza Naeem = conception of the study, drafting of the work, final approval and agreeing to the accuracy of the work.

## Consent

This study was not done on patients or volunteers, therefore no written consent was required.

## Registration of research studies

1. Name of the registry: Not applicable
2. Unique Identifying number or registration ID: Not applicable
3. Hyperlink to your specific registration (must be publicly accessible and will be checked): Not applicable

## Trail registry number

Not applicable.

## Guarantor

Hamna Raheel, Aiman Rahim, Unaiza Naeem.

## Declaration of competing interest

None.
